# Adaptive Tele-Therapies Based on Serious Games for Health for People with Time-Management and Organisational Problems: Preliminary Results

**DOI:** 10.3390/ijerph110100749

**Published:** 2014-01-07

**Authors:** Maite Frutos-Pascual, Begoña García Zapirain, Amaia Méndez Zorrilla

**Affiliations:** DeustoTech Life (eVIDA), Faculty of Engineering, University of Deusto, Bilbao 48007, Spain; E-Mails: mbgarciazapi@deusto.es (B.G.Z.); amaia.mendez@deusto.es (A.M.Z.)

**Keywords:** ADHD, tele-therapy, time management, Serious Games, intelligent systems

## Abstract

Attention Deficit with Hyperactivity Disorder (ADHD) is one of the most prevalent disorders within the child population today. Inattention problems can lead to greater difficulties in completing assignments, as well as problems with time management and prioritisation of tasks. This article presents an intelligent tele-therapy tool based on Serious Games for Health, aimed at the improvement of time management skills and the prioritisation of tasks. This tele-system is based on the use of decision trees within Django, a high-level Python Web framework. The technologies and techniques used were selected so as to boost user involvement and to enable the system to be easily customised. This article shows the preliminary results of the pilot-phase in an experiment performed to evaluate the use of adaptive tele-therapies within a group of typically developing children and adolescents aged between 12 and 19 years old without ADHD. To do so, we relied on the collection of parameters and the conduct of surveys for assessing time management skills, as well as measuring system usability and availability. The results of a time management survey highlighted that the users involved in the trial did not use any specific or effective time management techniques, scoring 1.98 and 2.30 out of 5 points in this area for ages under 15 and over 16 years old, respectively. The final calculations based on the usability questionnaire resulted in an average score of 78.75 out of 100. The creation of a customisable tool capable of working with different skills, in conjunction with the replication of the current study, may help to understand these users’ needs, as well as boosting time management skills among teenagers with and without ADHD.

## 1. Introduction

Attention deficit with hyperactivity disorder (ADHD) is one of the most prevalent disorders within the child population today, affecting an estimated 5.29% of children worldwide [1]. At present the diagnosis and treatment of ADHD should be performed in a multimodal environment in which information is provided to parents, teachers and patients with ADHD. In addition, the latter must have psycho-educational support at school, as well as psychological support in cases where it is strictly necessary (for the individual, the family and the community) and pharmacological treatment, if required by the specific symptoms [2]. Over-diagnosis must be minimised and a strict control of the administration of medication must be ensured [3].

ADHD usually manifests before the child is 7 years of age and is characterised by a certain degree of impulsiveness, inattention and excess of activity which does not match the child’s developmental age and is not appropriate in all situations that may arise [4]. In addition to these hyperactivity and inattention problems, organisational difficulties such as time management may also occur, especially within the school setting [5], as well as in adulthood, in the individual’s professional life.

Time management is meant as the ability to use the resource of time in the most efficient way. This efficiency depends upon task management and prioritisation, routine planning and the ability to remember what is still to be done. These management, organisation and perception-related problems are detailed in the current definitions of ADHD [6,7,8]. Toplak *et al*., in their review of the literature, reported the most relevant features associated with these management problems. These include delays in the performance of specific tasks, problems related to turn-taking and premature answers [9], as well as difficulty in prioritising tasks.

Time management and the estimation of the likely duration of tasks differ among the users diagnosed with ADHD and those belonging to the control group, both in adulthood and in childhood. This difference has been reflected in many studies, especially by assessing the estimation of the length of short periods of time while performing specific psychological tests [10,11,12,13,14,15].

Parents, doctors and teachers all report these types of behaviour, especially when they are linked to a lack of organisation and the acquisition of the appropriate time-planning strategies [16]. Organisational problems such as the ones derived from poor planning of an academic course or a subject, lead to bad academic results that can end in school failure in the most dramatic cases.

It is important to work on these management skills from childhood, providing the tools and the knowledge necessary to empower users. The improvement and promotion of these skills is directly connected, in most cases, to an improvement in school performance [17].

Succeeding in motivating a child affected by ADHD to undertake academic activities is a key element in their development. There is a need to encourage their learning process and memory, concentration and time management skills using structured activities, clear rules and striking materials. The possible solutions or improvements include developing the interest of the group towards these activities, which, together with an early and continuous intervention [18], can achieve a behavioural reduction of these problems and the negative consequences that arise in the mid- to long-term.

The use of techniques employed in the design of videogames, combined with the establishment of certain goals, guidelines and rules which not only encourage the use of these type of technologies, but also serve to reinforce the training of such skills as working memory, stimulation of attention, concentration and the aforementioned management skills, represent a new type of effective therapy in their application to ADHD [19,20].

The online performance of these therapies provides the users involved with greater autonomy, fostering communication between participating groups (doctors, psychologists, parents, teachers, and children, among others) [21]. Moreover, tele-therapies can be used and implemented on a simultaneous basis, and they are available in different places and on different devices [22].

The aim of this article is to assess the time management abilities of a group of typically developing children and adolescents, and to make a preliminary evaluation of the utility, usability and availability of the tool with respect to that group. These tests are intended to serve as guidance for adapting the tool presented in this article to final users, helping them to evaluate whether it could be used on a daily basis with users with and without ADHD. An important additional aim of the present study is to discuss whether the use of online game-based interventions fosters motivation and engagement in therapies. A supplementary objective is the evaluation of the use of online therapies to promote availability and convenience while using the tool. A final and future objective is to further assess the impact on the mid-term usage of this tool in teenagers with ADHD.

To accomplish the stated aims, this article presents an online interactive tool aimed to assist in the prioritisation of tasks by children and adolescents with ADHD, based on the techniques used in Serious Games for Health. This tool has been designed to strengthen time management skills related to day planning, and to help parents and instructors to determine which areas cause more confusion when establishing organisation strategies. The ultimate goal of this application is to serve as guidance to final users, providing them with advice and recommendations about management, as well as giving them sufficient autonomy to plan their day, by prioritising certain tasks above others.

In Section 2, the use of games for health in the diagnosis and treatment of ADHD will be placed in context, and the use of time management and organisation strategies in people with ADHD will also be studied. Subsequently, materials and method sections will be introduced along with the results, and followed by a final discussion and conclusion.

## 2. Background

This section reviews the previous work available in the literature on the use of Serious Games for Health in the field of ADHD, as well as recent studies on the evaluation of time management skills in people with ADHD.

### 2.1. Serious Games for Health and ADHD

This section analyses the use of Serious Games for Health in the diagnosis and treatment of ADHD. These games are specially designed to evaluate, diagnose, and be employed in therapies with children affected by ADHD, and have captured the attention of schools in recent years. The reason for this interest lies in the fact that many children who do not inhibit their hyperactive and/or impulsive behaviour, are capable of regulating it while playing videogames that motivate and foster their concentration [23,24].

It also should not escape notice, especially in relation to commercial videogames, that there seems to be evidence concerning the vulnerability of certain subgroups diagnosed with ADHD to videogame addiction [25,26]. Nonetheless, recent studies show that there is no direct link between the exposure to the use of videogames and attention problems, but there are other risk factors such as an inadequate family environment or anxiety problems [27].

#### 2.1.1. Diagnosis and Evaluation

This section details the most relevant publications related to the diagnosis and evaluation of ADHD using Serious Games for Health. A game designed and tested to help in the diagnosis of ADHD called *The Supermarket Game* has been developed in Brazil. It has been tested with 80 children diagnosed with ADHD and it has proven to be an efficient way of distinguishing among children who have, and who have not been diagnosed with ADHD. Decision-making algorithms are currently being optimised in order to discriminate among different types of ADHD [28,29]. Several years earlier, Rizzo, Bewerly *et al*. designed a 3D virtual classroom to help in the diagnosis of ADHD based on the interaction with the system. This system was tested with eight children with ADHD and 10 non-diagnosed children with positive and accurate results [30]. The concept of the virtual classroom was also used in the AULA Nesplora project, which implemented a version of Conners’ Continuous Performance Test using a 3D virtual classroom. It was initially tested with 57 children and it is currently being used in some Spanish assessment environments [31].

*Cyber Cruiser* is a car rally-style game oriented towards the assessment of the executive function and prospective memory in children with ADHD, and has been used to establish differences in these skills among diagnosed and control groups in a sample of 80 children [32], with positive results as assessed by the Conners’ Parent Rating Scale [33]. The improvement of executive memory has undergone several developments based on games over the past few years. The most current one is the design and implementation of two sets of Serious Games, *Cognitive Carnival* and *Caribbean Quest* in 2012 [34,35].

#### 2.1.2. Treatment

At present, there exist some evidence indicating that videogames can contribute to the improvement, regulation and standardisation of some symptoms related to ADHD. The first therapeutic videogame available to do so was developed by Pope and Bogart in 1996, and is a modification of software developed by NASA for the training of pilots, based on the progressive adaptation of a computer programme to user attention levels [36]. In 2001, Pope and Palsson went further and carried out a broader development of this patent as an intervention for the improvement of ADHD by modifying commercial videogames in combination with the use of the measurements obtained by an EEG [37].

Another alternative is *The Journey to Wild Divine*, a 3D virtual world created to be controlled through biofeedback, and available for purchase. This game is controlled by relying on relaxation techniques, which are very useful in the regulation or monitoring of hyperactivity and impulsivity in children and adolescents with ADHD [38]. At the end of 2012 a study started in London to evaluate self-management skills in 25 children with ADHD, in which a virtual helicopter was controlled by using Images by Magnetic Resonance (IMR) [39] to measure the activity on certain areas of the brain. In 2008 a game called *Self City* was released. It is oriented towards the improvement of social skills in adolescents diagnosed with ADHD and/or Pervasive Developmental Disorder. This game was developed in the shape of a 3D virtual world in which the adolescent is forced to cope with several situations [40].

The games not specifically designed for ADHD, but oriented towards a wide spectrum of disorders in children include *Play Mancer* and *Personal Investigator* (*PI*). *Play Mancer* emerged from a European Cooperation Project in 2007, aimed at the creation of a common framework for the development of therapy-oriented Serious Games [41,42]. *PI* is a 3D game specially designed to help adolescents with disorders through the use of Focused Solution Therapies—SFT and therapy games. It was initially tested with 4 adolescents with positive results [43].

In addition to the games specifically designed for the treatment and/or diagnosis of ADHD, among another disorders, commercial videogames adapted to ADHD therapy programs have also been used over the past few years, including *Robomemo* (CogMed, Stockholm, Sweden) [44]. Table 1 shows a summary of the results obtained.

Building on the background developed in this section along with the information available in Table 1, the authors would like to highlight several projects classified into two different groups: Number of users involved; and systems used outside of a laboratory setting.

Due to the number of users involved in the testing of the project, which strengthens the systems described, *The Supermarket Game* and *CyberCruiser* can be featured. These two projects have been tested with over 50 users.

The next group of projects have been selected due to their use outside of a laboratory setting. In this section *Aula Nesplora, The Journey to Wild Divine* and *Play Attention* need to be considered. These three systems are currently being used outside of the laboratory by professionals in psychotherapeutic or scholastic settings (*Aula Nesplora* and *Play Attention*). *The Journey to Wild Divine* is a commercial title available for online purchase.

**Table 1:** Review of Serious Games and ADHD.

Authors	Game Name	Year	Country	Type	Goal	Technologies
Pope and Bogart [36]	-	1994	US	Development	Fostering attention	EEG, PC
Peter Freer [45]	*Play Attention*	2000	US	Development/In Use	Fostering attention	EEG, PC
Kerns [32]	*Cyber-Cruiser*	2000	CA	Development	Evaluating executive functioning and prospective memory	PC
Pope and Palsson [37]		2001	US	Commercial videogame adaptation	Fostering attention	Play Station, EEG
Bell, Smith *et al.* [38]	*The Journey to Wild Divine*	2003	US	Commercial	Relaxation, Mindfulness	Biofeedback, PC
Rizzo, Bewerly *et al.* [30]	*-*	2004	US	Development	ADHD diagnosis	3D virtual classroom, PC
Coyle, Sharry *et al.* [43]	*Personal Investigator*	2005	IE	Development	Focused Solution Therapies	Virtual world, PC
Andrade *et al.* [28,29]	*Supermarket game*	2006	BR	Development	ADHD diagnosis	PC
Conconi, Jiménez *et al.* [41,42]	*Play Mancer*	2007	EU	Development	Creating a common framework for Serious Games-based therapies	Virtual world, PC
Van Dijk, Hunneman *et al.* [40]	*Self-City*	2008	NL	Development	Fostering social skills	PC
Bartle [34,35]	*Cognitive Carnival*	2012	CA	Development	Executive memory	PC
Bartle [34,35]	*Caribbean Quest*	2012	CA	Development	Executive memory	PC
Rubia *et al.* [39]	*-*	2012	GB	Development	Self-control	MRI, Virtual helicopter
Díaz-Orueta, García-López *et al.* [31]	*AULA Nesplora*	2013	ES	Development/In Use	Testing attention: Conners’ CPT	3D virtual classroomPC

**Table 2:** Review of Time Management and ADHD.

Authors	Year	Country	Participants	Method	Assessment	Result
Abikoff, Gallagher *et al.* [46]	2013	US	158 Children: 64 Organisational Skills Training 61 Precluded Skills training 33 Wait-List Control	20 individual clinic-based sessions 10–12 weeks	1 month post-treatment 2 in the following school year Measures: Organizational Skills Training (OST) Children’s Organisational Skills Scale for Parents (COSS-Parent) Children’s Organisational Skills Scale for Teachers (COSS-Teacher)	Promises clinical utility in children with ADHD and organisational deficits
Langberg, Becker *et al.* [47]	2013	US	23 Children	Homework, Organisation and Planning Skills Intervention (HOPS) Included demographic and child characteristics	Parent-rated materials organisation and planning skills	Importance of teaching students with ADHD to use structured organisation systems
Pfiffner, Villodas *et al.* [48]	2013	US	57 Children (mean age 8.1 years) 17 girls 40 boys	Collaborative Life Skills Programme (CLS Programme) 10 schools 3 integrated components over 12 weeks	-	Support the focus of CLS on both ADHD symptom reduction and organisational skills improvement
Parker, Hoffman *et al.* [49]	2013	US	19 students	10 different US campuses One-on-one interviews	Learning and Strategies Inventory	ADHD helped participants to enhance their self-control
Field, Parker *et al.* [50]	2013	US	160 college students: 70 Female 90 Male	Weekly phone-based coaching interviews	Learning and Study Strategies Inventory (LASSI) College Well-Being Scale	Statistically significant higher total scores in both scales
Hart, Radua *et al.* [51]	2012	UK	150 patients 145 healthy controls	Peak coordinates extracted from: Case-control activation differences Demographic, clinical and methodological variables	Meta regression analyses	Suggests potential normalisation effects on the function of the pre-frontal region with long-term psycho-stimulant treatment
Bioulac, Lallemand *et al.* [52]	2012	US	36 boys 20 ADHD 16 controls	Virtual classroom task	Continuous Performance Test (CPT)	Children with ADHD are vulnerable to time-on-task effect on performance
Langberg, Epstein *et al.* [53]	2012	US	37 middle school students with ADHD 24 HOPS intervention 13 wait-list control	HOPS Intervention 3-month follow-up	Parent and teacher ratings of organisational skills and homework problems were collected. School grades were also collected	Intervention participants did not make significant improvements relative to the comparison group according to teacher ratings
Gureasko-Moore, DuPaul *et al.* [54]	2008	US	3 male students enrolled in a public secondary school	Training in self-management procedures.	-	Results were consistent across the 3 participants.

By examining Table 1 it can be seen that very little work on time managment skills has been done, the analysed references being mostly focused on attention problems. As was shown in Section 1, the literature has established that developing these management skills constitutes a key factor for children and teenagers diagnosed with ADHD, due to their issues with estimating and prioritising tasks. Upon review of the literature, it followed that there is clearly a need to work on these problems effectively, which resulted in the solution presented in this article.

This solution is focused on techniques used in the field of Serious Games, combined with work on time management skills, particularly centred on prioritising tasks. Furthermore, the fact that these exercises can be carried out online makes this tool accessible anywhere, and could serve as a consultation tool in everyday life.

### 2.2. Time Management and Organisational Skills in ADHD

This section reviews the latest available interventions that are focused on the enhancement of time management skills and organisational behaviour in children and teenagers with ADHD. People diagnosed with ADHD may express organisational and time estimation problems [55,56]. Upon review of the literature, several authors have centred their attention on working and improving these skills with children and teenagers with ADHD. The following paragraphs review the latest studies about time management and organisational skills intervention.

Abikoff and his colleagues have been working for the last decade on the analysis and impact of various approaches to organisational capacities and time management skills on children with ADHD. This paragraph outlines some examples of their research. In 2003, Abikoff, Gallagher *et al*. performed an assessment, analysis and treatment of time management skills and planning deficits in children with ADHD [57]. Later, in 2009, Abikoff, Nissley *et al*. evaluated the effect of medication (methylphenidate-osmotic-release oral system [MPH-OROS]) on Organisation, Time Management and Planning (OTMP) interventions. Results showed that some children remained resistant to treatment. In 2013 they concluded that the importance of performing OTMP interventions with children with ADHD was due to the promising clinical results [46].

In 2013, Langberg, Becker *et al*. demonstrated the importance of teaching organisational and academic planning and management guidelines to children with ADHD. They administered a Homework, Organization and Planning Skills (HOPS) intervention in a school setting [47]. In the same year, Pfiffner, Villodas *et al*. developed a new school-home collaborative intervention (Collaborative Life Skills Program‒CLS) for youngsters with attention and/or behavioural problems. This study concluded that there was a reduction of symptomatology related to ADHD and there was an enhancement of organisational abilities [48]. Parker, Field and other colleagues published several articles identifying undergraduate students with ADHD perceptions and needs that were related to academic coaching. Participants provided positive feedback about academic coaching, as they perceived an improvement in their performance and self-organisation abilities [49,50].

In 2012, a considerable number of studies were published on the implications of time management and estimation deficits in people with ADHD. Hart, Radua *et al*. assessed time estimation abilities in people with ADHD by performing the meta-analysis of fMRI images. They found potential normalisation effects in pre-frontal region linked with the use of long-term psycho-stimulant treatments [51]. In this same year, Bioulac, Lallemand, Rizzo *et al*. evaluated the use of a 3D virtual classroom and concluded that children are vulnerable to a time-on-task effect on their performance. They stated that virtual reality is a reliable method to test ADHD children’s ability to sustain performance over time [52]. Prior to the research published in 2013, Langberg, Epstein *et al*. studied the use of HOPS intervention with a 3-month follow-up parent-rated organised action, on planning and homework completion behaviour. They stated that participants made significant improvements relative to the wait-list comparison, taking into account parent-rated assessment, while participants did not make significant improvements relative to the comparison group according to teacher ratings [53].

In previous years other references can be found in the literature to the use of specific interventions for people with ADHD focused on time management skills. In 2008, Langberg, Epstein *et al*., underlined the need to perform controlled studies on the use of specific interventions aimed at fostering executive functions [17]. In 2006, Gureasko-Moore, DuPaul *et al*., engaged in the controlled monitoring of a small group of secondary students with ADHD, which showed an improvement in the use of organisational techniques in a school setting [54].

Table 2 outlines the main characteristics of each of the studies included in this review, with specific information about experiment setting, results and participants’ description. Please refer to this for a further analysis. As described in this section and also stated in Table 2, previous studies show the efficacy of performing specific interventions with children and teenagers with ADHD. Nevertheless, the authors have found that there is currently a lack of support tools to help these users in their day-to-day lives, beyond specific interventions taking place at a specific time.

The authors have identified a need to develop tools that are fully available online at any time of the day. These tools should be ready to help and assess users outside of the specific interventions. That is why it is interesting to develop tools intended to complement the interventions focused on time management. These developments should be aimed at answering specific inquiries or doubts that may eventually arise. The system developed fits this description, as it provides final users with a consultation tool that fosters the autonomous self-management of time.

## 3. Materials and Methods

This section shows the materials and methods used in the development and testing of the online application discussed in this article. The tool consists of a virtual interactive balance which, by making use of decision trees and user-entered parameters, is capable of prioritising between two proposed activities.

### 3.1. Participants

Preliminary evaluation of the tool was made with a group of typically developing children and adolescents aged between 12 and 19 years old, with an average age of 16.23 years old.

Seventeen randomly selected participants (seven women and 10 men), selected from a group of volunteers, took part in these trials. These users are resident in the Basque Country, Spain, have not been diagnosed with ADHD and have Spanish as their mother tongue. For individuals under 18 years old the approval of parents or guardians was requested prior to conducting the surveys.

Users responded to questionnaires and tests independently, and used their own online devices (mobile, tablet and/or smartphone) to do so. Participants were allowed to choose the time of day when they wanted to perform the evaluation of the tool. This evaluation took place during the months of July and August 2013.

### 3.2. Methods

This section details the technical features of the system and describes the questionnaires delivered to the users before and after using the application. The online tool was produced in Django [58], the high-level Web framework for Python [59]. The results obtained and the necessary parameters were stored in a MySQL database [60]. User interface and user interaction were developed using JQuery [61], Javascript and CSS, with Touch Punch being used to adapt the tool to touch screens [62]. The priority activity decision algorithm was calculated by using a decision tree implemented in Python. The technologies and techniques employed here were selected in order to boost user-involvement and the adaptation of the system to users’ needs.

For the evaluation of users’ time management skills prior to the test, the *Time Management Behavior Questionnaire* (hereinafter TMBQ) by Macan *et al.* [63,64,65] was used, adapted to the Spanish language. This scale was chosen for its prestige and validity in the measurement of time management skills, especially for the age range selected in the test. The Spanish version was validated and accepted [66,67]. The aim of this questionnaire was to learn about and analyse the management skills of the participating users. It is composed of 34 items divided into four sections, as shown in Table 3. Items were evaluated using a Likert scale ranging from 1 (“never”) to 5 (“always”). This questionnaire was available online, by means of the Google Forms tools [68] during the months of July and August 2013.

**Table 3:** TMBQ—Sections [66].

Section	Interpretation
**F1**	*This section describes students’ willingness to prioritise and select tasks, in order to obtain specific goals. High scores in this section show an effective task prioritisation.*
**F2**	*Area 2 contains the items related to the use of specific techniques associated with effective time management. High scores on this section indicate effective time management skills using specific techniques.*
**F3**	*This section evaluates the way subjects organise their time, and how their study environment is structured. High scores in this section indicate a preference for a disorganised environment.*
**F4**	*This area shows the degree of control users perceive to have over their own time. High scores in this area indicate a high control over their time.*

The discussed tool was evaluated by a user satisfaction test based on the *System Usability Scale* [69] (henceforth *SUS*). This questionnaire consists on 10 items. These items were evaluated by using a Likert scale ranging from 1 (“strongly agree”) to 5 (“strongly disagree”). The completion of the questionnaire aims to continue to adapt the system to the users’ final needs. This questionnaire was available online, by means of the Google Forms tools during the months of July and August 2013.

Scoring for the SUS was as follows: each item is ranged from 0 to 4. For items 1, 3, 5, 7 and 9, the score contribution was scale position minus 1. For items 2, 4, 6, 8 and 10, the score contribution was 5 minus the scale position. The sum scores were multiplied by 2.5 to obtain the overall value for the SUS [69].

### 3.3. Experiment Description

**Figure 1:**
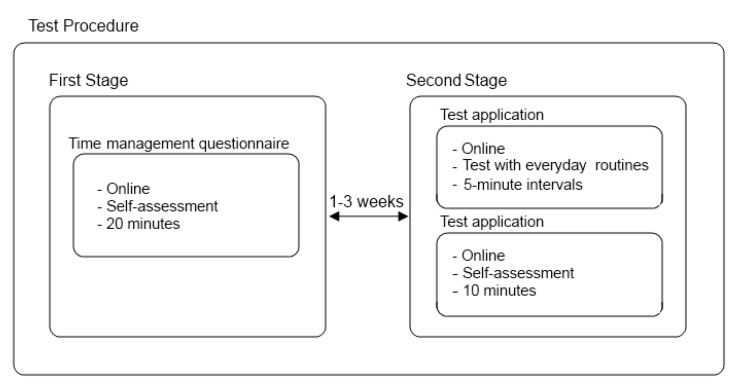
Test procedure.

The tool’s complete testing procedure was carried out online, outside of the laboratory environment, and in the course of users’ everyday life. These conditions were considered appropriate due to the nature of the system. Each user answered the questions and tested the tool on their own device, in order to verify the accessibility of the system and whether it fulfilled the criterion of being multi-platform. Figure 1 shows the procedure followed during tests, with the time interval left between the two testing stages.

As shown in Figure 1, the system’s testing procedure was carried out in two mutually independent stages, between one to three weeks apart, on the basis of the results obtained in the first stage and adapting it to users’ availability.

The first stage involved conducting a questionnaire about time management habits. This questionnaire was prepared online, making use of Google Forms. It consisted of 34 items divided into four different areas, each one focused on a specific skill (effective time management, preference for disorganisation, establishing concrete objectives and perception of time). Items were evaluated by using a Likert scale ranging from 1 (“never”) to 5 (“always”). The purpose of this analysis was to establish the global situation of the user in terms of time management skills. This test took around 15 to 20 minutes to complete.

The second stage was comprised of two sections: firstly, the use of the application, and secondly, the performance of a systematic evaluation of the questionnaire. The application test was conducted in the course of a full day, integrating it in a natural way into the users’ routine. This testing required various easy questions in the application, on the basis of the activities carried out. It took between 15 to 30 min, depending on the number of attempts users made.

Once this battery test was finished, the users took the online usability questionnaire *SUS* [69], translated into Spanish. This usability questionnaire was composed of 10 items evaluated by a Likert scale ranging from 1 (“totally disagree”) to 5 (“totally agree”) and aimed at measuring system usability. This survey was conducted in order to prove if the design and purpose of the system was easy to learn and user-friendly. This test took around 5 to 10 min to complete.

All the questionnaires and tests of the application were carried out in an autonomous way by users, outside of the laboratory environment. The two testing stages were carried out on their own devices, adapting the tests to the aim of the application; they took between 35 min to 1 h to be completed successfully. The results obtained in these two stages were uploaded and analysed in the *IBM*—*SPSS Statistics* [70] predictive analysis software tool*.*

## 4. Results

This section shows the technical results of the system, and the ones obtained after the user- experience evaluation process.

### 4.1. Technological Results: Tele-Therapy System

In this section the application design and development is explained. The application is an online platform developed in Django, which aids in the improvement of time management skills, and more particularly, in task prioritising.

A tool was designed which consists of three parts: training, consultation and monitoring. All of them are subject to the same format, but their purposes differ; each one of them will now be explained in detail.

*a Tool for queries*

**Figure 2:**
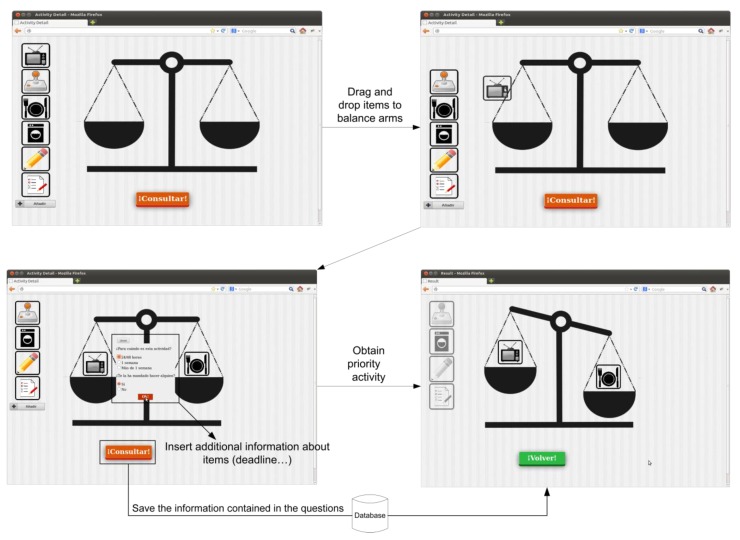
Tool for queries flow.

The consultation tool is a virtual balance in which users drag two activities that cause confusion by means of drag & drop techniques. Figure 2 shows the flow diagram of this tool for queries. 

In addition to dragging the activities, as it is shown in Figure 2, they have to enter relevant information about the activities, such as the deadline for performing them, and, in the case of specific activities, they have to report whether someone (father, mother or someone else) asked them to do the activities.

**Figure 3:**
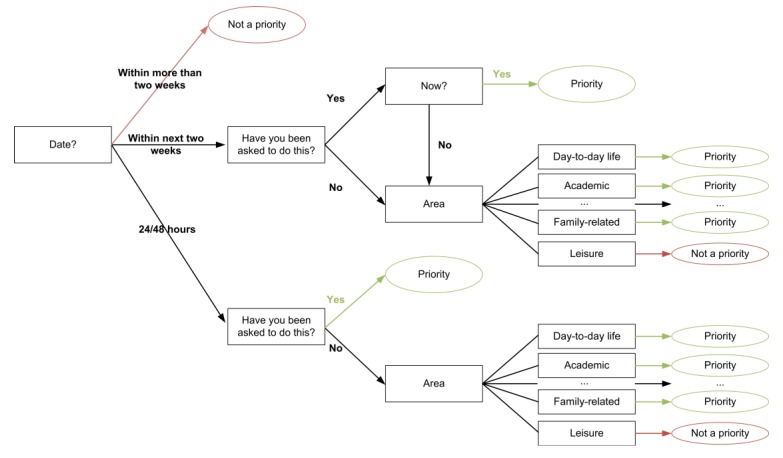
Implemented Decision Tree.

As it is shown in the decision tree in Figure 3, a decision can be made on the basis of whether an activity needs to be a priority or not. Each branch of the tree is associated with a specific and unique weight. If two activities are labelled as a priority, the system decides, according to which one of them has a greater weight, which is the high-preference activity.

The decision tree was made following the ID3 algorithm [71], which determines how to divide the information and when to stop dividing it [72]. In order to decide which feature is the first one to divide the tree, Shannon entropy [73] was used, defined by Equation (1):

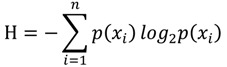
(1)


The tool is fully configurable, and allows the user to add new activities to the system by entering a series of parameters. This way it is possible to scale the size of the system and cover new application activities and areas. The final purpose is to serve as a consultation system when working on task prioritising skills.

On the basis of these parameters and of the area in which the tasks are classified, the system is responsible for evaluating them, and determining which one needs to be a priority by making use of the decision tree shown in Figure 3.

*b Tool for training*

In the training tool the user is presented with two activities already placed in the balance, with a series of features associated with each one of them (execution date, whether or not it has been requested by someone, among others). The user must discern, using the data provided, which activity needs to be a priority.

**Figure 4:**
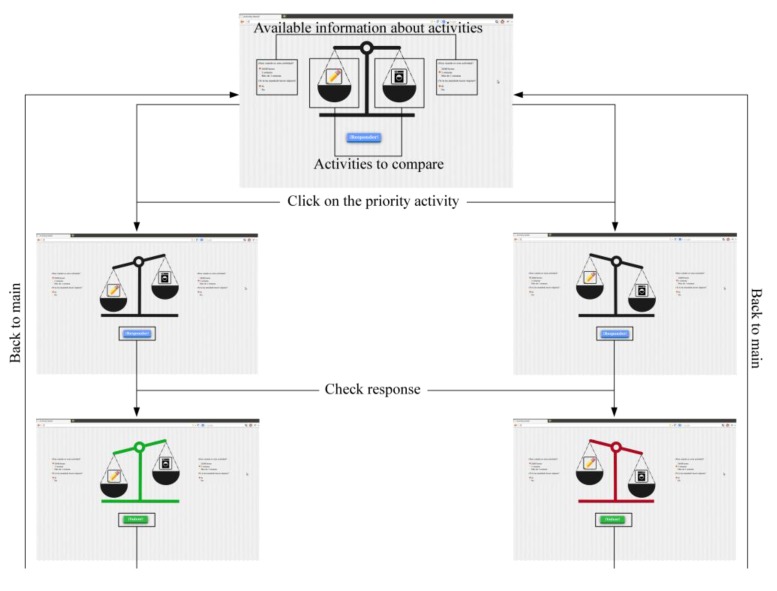
Tool for training diagram.

This game mode was developed in the way of a “quiz”, where users continue working on the task prioritising on the basis of the area (academic, personal, family, leisure, among others) and the given parameters. Figure 4 outlines the flow diagram of the tool.

Figure 4 shows the flow diagram of the “Quiz” section. The activities are customised by means of the queries the user may make, adapting automatically to those areas that cause the most difficulty.

*c Tool for supervisors*

The supervisor tool keeps a history of all the queries made and the results obtained by each user from the training tool, showing the questions with their answers, together with the date of completion, in order to determine which areas are most difficult for each user. Furthermore, this tool allows users’ exercises to be customised, enabling questions to be added to the tool concerning the specific working skills of each user. This way the system is not only adapted to the exercises contained in the tool, but it is also fully customisable from the supervision area.

### 4.2. User Experience Evaluation

This section describes the preliminary results of the tool shown in this article. These results were taken during the months of July and August 2013. The tests consisted of three parts: completing a questionnaire in Spanish about time management skills, performing some exercises by using the online tool, and finally, answering an online questionnaire about the usability of this tool.

**Figure 5:**
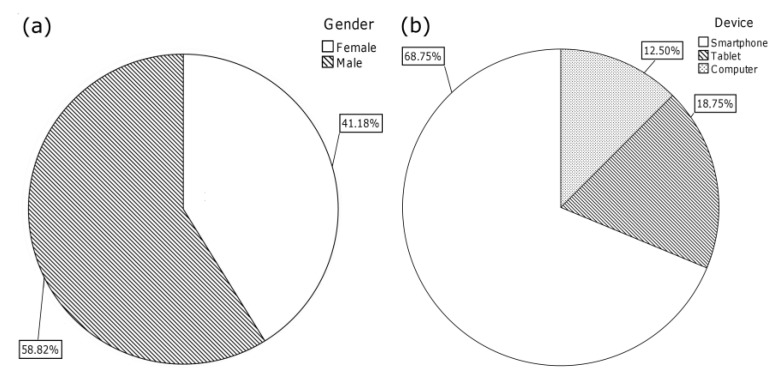
(**a**) Gender segmentation; (**b**) Device segmentation.

Participants were 17 users randomly selected from a group of volunteers. These users performed the referred tests on their online devices in the summer of 2013. Seven women and 10 men participated in these tests, as shown in Figure 5a.

Figure 5b shows the segmentation of devices among users during the tests. Participants had the flexibility to choose the device and platform on which to perform the tests, and the only requirement was an Internet connection. The majority of the group chose to use their smartphones and only five participants decided to use other devices, such as a computer (two users) or a tablet (three users).

In order to thoroughly analyse the results, the sample was divided into two age-groups. Due to the number of users that took part in this study, a Mann-Whitney non-parametric test was applied. This test was significant (*p* < 0.05) for the 12–15 year-old and the 16–19 year-old groups. The following sections describe each of the tests, along with the results obtained.

*a Test 1: TMBQ*

**Figure 6:**
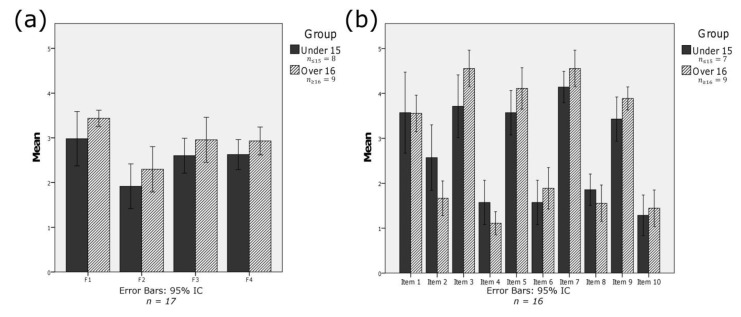
(**a**) TMBQ—responses; (**b**) SUS questionnaire—responses.

The evaluation of participants’ time management skills was done by using a Spanish validated version of the *TMB Questionnaire* [66]. This questionnaire consists of 34 items divided into four different areas; please refer to Table 3 for details. Items were evaluated by using a Likert scale ranging from 1 (“never”) to 5 (“always”). Average results obtained in each of the areas described are those shown in Figure 6a. These mean values were obtained by calculating the average of each of the items within each area and the responses of the 17 participants divided into two groups.

As shown in Figure 6a, the average rating per block varies between 2.98 and 1.92 points, out of a total of 5, for the group under 15 years old and between 3.44 and 2.30 for the group over 16 years old. The areas that make these extremes are those corresponding to the willingness of students to set specific goals (area 1; corresponding to F1) and the use of specific effective techniques in time management (area 2; corresponding to F2).

*b Online tool evaluation*

The section of the tool used for evaluating user experience is available online [74]. This is a multi-platform tool which allows participants to select when and how they want to perform the tests.

**Figure 7:**
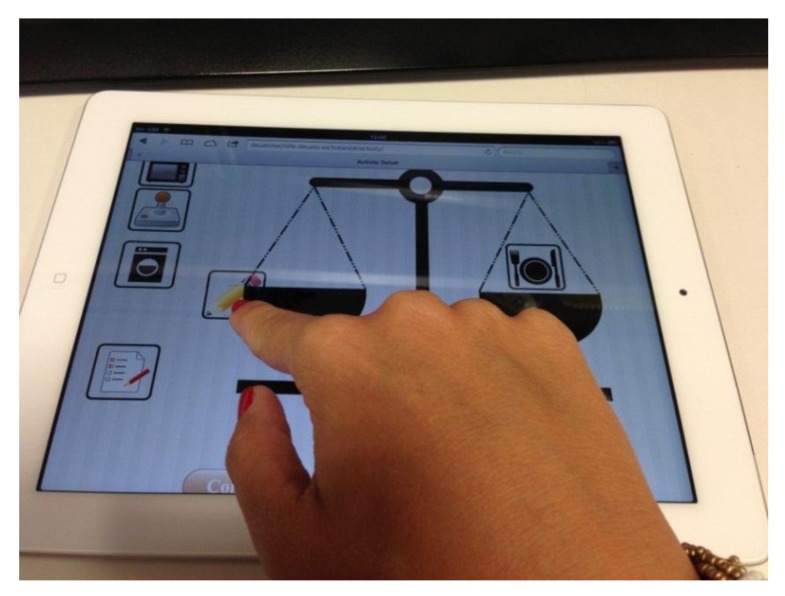
Online tool using a tablet device.

Figure 7 shows one of the seventeen participants performing the usability evaluation of the described tool from a tablet device.

*c Test 2: SUS Questionnaire*

In order to evaluate system usability, the SUS questionnaire [69] was used, which consists of 10 items. The link for the completion of this questionnaire was provided to users alongside with the link to the online tool. Items were evaluated by using a Likert scale ranging from 1 (“strongly disagree”) to 5 (“strongly agree”).

As far as the answers are concerned, 16 participants, seven women and 9 men, out of the 17 initial participants in the trial, answered the system usability questionnaire. Due to schedule issues, one of the participants left the testing after the completion of the first questionnaire. This user belonged to the group under 15-years old. The inclusion of this user in the first test was not considered decisive in order to obtain the results. Please refer to Figure 6b for average answers per item obtained for each age-group. The SUS mean overall score was 78.75 out of 100, with a standard deviation of 6.39. Users under 15-years-old scored an average result of 73.93 out of 100, with a standard deviation of 4.97 while participants over 16-years-old rated the system with an average result of 82.5, with a standard deviation of 4.68.

## 5. Discussion and Conclusions

This article addresses the issue of time management and the prioritisation of tasks in teenagers with and without ADHD diagnosis. In this section, authors attempted to answer the following questions, drawn from the section of this article which set out the objectives: Is it possible that the use of interactive and personalised content, such as serious games, encourage the use of tools to improve time management skills? Does the development of online tools foster their availability? Do users feel comfortable using these tools?

Mobile devices are increasingly used in daily life, relegating computers to the background when it comes to short, specific activities [75]. Results obtained in this study show that 15 out of 17 participants used mobile devices to perform the activities proposed in this trial. The rise in the use of these devices, along with access to a permanent Internet connection, increases the demand for interactive online content, enabling the emergence of tools like the one presented in this article.

According to the literature mentioned in Section 1 and Section 2, serious games within these interactive tools can also help to improve specific skills through the use of gaming techniques and appealing and accessible content.

As explained in Section 1, time management skills and task prioritisation capabilities need to be developed during childhood and adolescence, in order to prepare users for adult life and the management of their own time. Moreover, they could also be key skills in obtaining good academic results.

Based on the review conducted by the authors in Section 2.2 about the strengthening of time management and organisational skills within the ADHD collective, it is clear than specific approaches and measurements should be taken. After analysing the literature, it can be determined that there is an agreement about the efficiency of targeted face-to-face therapies focused on the enhancement of time management skills [46,47,48,49,50,51,52,53,54].

Bioulac, Lallemand *et al.* proposed a virtual development that implied the first steps in the evolution of face-to-face therapies to more innovative interventions. These new therapies included the use of new technologies such as virtual environments and computer-based therapies, thus providing users with a greater autonomy [52].

The described system presented in this paper goes one step beyond, by including the use of online therapies which promote availability and convenience while using the tool. These therapies serve as a supporting tool for children and adolescents with time management and organizational problems. The development of new online solutions could help to complement traditional therapies fostering their effects and helping to create efficient management and organisational skills.

Results obtained from the TMBQ show scores below 3.5 points out of 5 in every area. These results are especially low when it comes to the use of specific techniques for efficient time management skills.

These results suggest the need for the creation of new, efficient, and attractive content that provides guidance and effective techniques to work on time management skills. It is therefore necessary to create an accessible tool for time management habits and the prioritising of tasks for adolescents in general, and not only for those diagnosed with ADHD.

The described tool is focused on working on these activities, particularly on prioritising tasks. When analysing its usability, results were in 78.75 out of 100. Although this result means a good acceptance of the system, the authors have analysed its weaknesses in order to implement possible improvements. Segmenting participants by age shows that there are differences related to system usability and users’ comfort and confidence. Even though results were acceptable in both groups (please refer to Section 4 for details), the groups of 12–15 year-old users showed a greater divergence of views when they were asked to evaluate whether they felt comfortable with the system or not. These results suggest that some users may not feel comfortable with the use of tools that can be used to control their daily routines, leaving a record of their activities.

As a conclusion to this study, it was confirmed that there is a need for new interactive content in order to work on time management skills in teenagers with and without ADHD. Key skills to be worked on lie not only in the field of the prioritisation of tasks, but also in the effective utilisation of specific techniques to that effect. Nevertheless, authors consider that this kind of adaptive tele-therapies should be adopted as a support tool for traditional therapies, not as a substitute for conventional interventions.

Future directions for this study in the field of the design and development of the system are expanding the tool to include new skills, such as effective time organisation, that continues along the lines of the current tool. Moreover, the system should be adapted to be suitable for all age-ranges, trying to minimise divergences between them. Additionally, providing a greater degree of customisation to the tool may help to improve the acceptance by both age groups.

Finally, the main direction for the future is to replicate this study:

With a higher number of users.
With users with and without an ADHD diagnosis.
With the segmentation of participants into two age groups.
Developing a bilingual or trilingual tool that allows the study to be replicated in other national and international territories in where reported diagnosis of ADHD is significantly different from the Basque Country, Spain.
Performing a longitudinal study on the impact of online game-based tools on time management skills in teenagers with and without ADHD.

The creation of a completely new tool capable of working on different skills, in conjunction with the replication of the current study, may help to understand these users’ needs and boost time management skills among teenagers with and without ADHD.
